# Antimicrobial resistance in neonates with suspected sepsis

**DOI:** 10.5588/pha.21.0038

**Published:** 2021-11-01

**Authors:** S. K. Yadav, S. K. Agrawal, S. K. Singh, A. Giri, G. K. Singh, R. Ghimire, A. G. Stewart, K. L. Show, F. L. Moses

**Affiliations:** 1 Nobel Medical College and Teaching Hospital, Biratnagar, Nepal; 2 B. P. Koirala Institute of Health Sciences, Dharan, Nepal; 3 College of Life and Environmental Science, University of Exeter, Exeter, UK; 4 Department of Medical Research, Yangon, Myanmar; 5 Sierra Leone Ministry of Health and Sanitation, Freetown, Sierra Leone; 6 College of Medicine and Allied Health Sciences, University of Sierra Leone, Freetown, Sierra Leone

**Keywords:** antimicrobial resistance, neonatal sepsis, NICU

## Abstract

**SETTING:**

Nobel Medical College and Teaching Hospital, Biratnagar, Nepal.

**OBJECTIVE:**

To determine the pattern of antimicrobial resistance and hospital exit outcomes in neonates with suspected sepsis in a tertiary neonatal intensive care unit (NICU).

**DESIGN:**

This hospital-based cohort study was conducted to follow patients from January to December 2019. All identified cases of suspected sepsis were enlisted from hospital records.

**RESULTS:**

Sepsis was suspected in 177 (88%) of the 200 cases admitted in the NICU; 52 (29%) were culture-positive. *Pseudomonas* was the predominant organism isolated (*n* = 40; 78%), followed by coagulase negative *staphylococcus* (*n* = 12, 23%). Nine (17%) of the 52 isolates were resistant to the Access and Watch group of antibiotics, including some resistance to Reserve group drugs such as imipenem and linezolid. Most treated cases (*n* = 170, 96%) improved, although 7 (4%) left against medical advice.

**CONCLUSION:**

Most of the pathogens were resistant to WHO Access and Watch antibiotics and occasional resistance was observed to Reserve group drugs. Most sepsis was caused by Gram-negative bacilli. Improving turnaround times for antibiotic sensitivity testing using point-of-care testing, and a greater yield of culture-positive results are needed to enhance the management of neonatal sepsis.

## Abstract (FR)

**LIEU:**

Hôpital Universitaire Nobel Medical College, Biratnagar, Népal.

**OBJECTIF:**

Déterminer le profil de résistance antimicrobienne et les résultats au moment de la sortie d’hôpital chez des nouveau-nés avec suspicion de sepsis dans une unité néonatale de soins intensifs tertiaires (NICU).

**MÉTHODE:**

Cette étude de cohorte hospitalière a été réalisée pour suivre les patients de janvier à décembre 2019. Tous les cas identifiés de suspicion de sepsis ont été inclus à partir des dossiers hospitaliers.

**RÉSULTATS:**

Une suspicion de sepsis a été observée chez 177 (88%) des 200 cas admis en NICU ; 52 (29%) étaient positifs par culture. *Pseudomonas* était le micro-organisme prédominant (*n* = 40 ; 78%), suivi des staphylocoques à coagulase négative (*n* = 12, 23%). Neuf (17%) des 52 isolats étaient résistants aux groupes d’antibiotiques « dont l’utilisation est essentielle » et « à utiliser sélectivement », avec certaines résistances aux antibiotiques « de réserve » comme l’imipénème et le linézolide. L’état de la plupart des cas traités (*n*=170, 96%) s’est amélioré, mais sept (4%) nouveau-nés sont sortis contre avis médical.

**CONCLUSION:**

La plupart des pathogènes étaient résistants aux antibiotiques des groupes OMS « dont l’utilisation est essentielle » et « à utiliser sélectivement », et quelques résistances ont été observées à certains « antibiotiques de réserve ». La plupart des sepsis ont été causés par des bacilles à Gram négatif. L’amélioration des délais d’exécution des tests de sensibilité aux antibiotiques à l’aide de tests au point de service et un meilleur rendement des résultats positifs par culture sont nécessaires pour renforcer la prise en charge du sepsis néonatal.

Neonatal sepsis refers to generalised bacterial infection with systemic signs, and is diagnosed using a positive blood culture in the first 4 weeks of life.^1^,^2^ It is classified by time of occurrence of infection as early onset sepsis, i.e., bacteraemia in the first 72 h of life, and thereafter, as late onset sepsis.^3^

It is a life-threating illness and a major cause of neonatal morbidity and mortality worldwide.^3^ Global incidence is estimated to be 1.3 million cases annually, resulting in about 203,000 attributable deaths. Estimates that 2,202 neonates per 100,000 live births develop neonatal sepsis globally, with a mortality rate of 11–19% (242–418 deaths/100,000 live births), are derived from high-income country data.^4^ However, lowand middle-income countries, where data are limited, are most affected due to the frequency of infections and limited access to good healthcare.

Due to the non-specific nature of the signs and symptoms in the new-born, most cases of sepsis are clinically diagnosed, then treated with empirical broad-spectrum antibiotics. This use of antibiotics promotes antimicrobial resistance (AMR). Bedside tests are not available and empirical antibiotic treatment contributes to the increased prevalence of antibiotic-resistant organisms. Examples include methicillin-resistant *Staphylococcus aureus,* vancomycin-resistant *Enterococcus* and multidrug-resistant Gram-negative rods.^5^,^6^ At least 700,000 deaths occur annually due to drug-resistant diseases, which is projected to rise to more than 10 million deaths globally by 2050 if action is not taken.^7^

The spectrum of multidrug-resistant bacteria varies. In neonates, sepsis <48 h after birth is most often caused by Group B *streptococcus* (43%) and *Escherichia coli* (16–29%).^8^,^9^ For very low birth weight infants with early onset sepsis, the *E. coli* rate exceeds that of Group B *streptococcus* (5.1 vs. 2.1/1,000 live births).^9^ Late onset sepsis is mainly caused by Gram-positive bacteria (49%), most often coagulase-negative *Staphylococcus* (45%). Gram-negative late onset sepsis is less common (23%), but is associated with greater mortality in neonatal intensive care units (NICUs) (19–36%).^9^ Both forms of sepsis are related to birth weight and the location where the infection was acquired.^10^

The neonatal mortality rate in Nepal is 21/1,000 live births.^11^ Neonatal infection is the third most common cause of neonatal mortality (17%; 3.6/1,000 live births), after preterm birth complications (33%) and intrapartum-related events (23%).^11^

In Nepal, 10–42% of all the patients are prescribed antibiotics for therapeutic or prophylactic purposes without bacterial confirmation or susceptibility testing. Further data on AMR in Nepal are limited, possibly due to a lack of prioritisation and the absence of routine surveillance.^12^ An appropriate antimicrobial agent should be used for sepsis to prevent the development of multidrug resistance and reduce morbidity and mortality.^13^

Nepal has significantly more multidrug-resistant bacterial strains closer to health care sites.^12^ In one tertiary hospital, coagulase-negative *Staphylococcus* was the predominant Gram-positive organism isolated from both early and late onset sepsis cases (46.6%), followed by *S. aureus* (14.6%). Other Gram-negative multidrug-resistant bacteria were associated with early onset sepsis: *Acinetobacter* species (9.5%) and *Klebsiella pneumoniae* (7.7%).^1^

Antibiotics used in Nepali hospitals were found to be effective in curing only 50% of infections.^14^ Furthermore, AMR contributes to increased treatment costs, hospital stay, morbidity and mortality.^15^

In Nobel Medical College and Teaching Hospital (NMCTH), Biratnagar, Nepal, a public-private partnership tertiary care referral centre, sepsis is the third most common cause of admission in the NICU, after asphyxia and meconium aspiration.^16^ However, there is no information about the AMR pattern, particularly in relation to patient outcomes. In the present study, we described the bacterial profile and determined the antimicrobial resistance pattern and hospital exit outcomes in neonates with suspected sepsis in the NMCTH NICU.

## METHODS

### Study design

This was a hospital-based, cohort study conducted from January to December 2019.

### General setting

Nepal, in South Asia, is mainly located in the Himalayas, with an estimated population of 30.2 million.^17^ NMCTH is in Biratnagar, the second most densely populated and third most populous city of Nepal, with a population of 242,548 (2011 census; https://unstats.un.org/unsD/demographic/sources/census/wphc/Nepal/Nepal-Census-2011-Vol1.pdf).

Nepal is moving towards an integrated response to AMR. The National Action Plan, led by the AMR Steering Committee at the Curative Division of Ministry of Health and Population, recognises the National Public Health Laboratory (under the Department of Health Services) as the focal point for AMR activities.

### Specific setting

NMCTH, affiliated to Kathmandu University, has 911 beds, <40,000 admissions per year and a bed occupancy rate of ~80%. The average outpatient flow is ~1000/day. The NICU is well equipped, with 17 beds, a nursing staff: patient ratio of 1:2–3 and the highest volume referral for tertiary care in eastern Nepal.

### Laboratory testing

Blood samples from the neonatal ward/emergency department were collected in brain heart infusion (BHI) broth (1 ml blood in 5 ml of BHI broth). After 24 h culture, the sample was sub-cultured onto blood and MacConkey agar (aerobic, 35°C) and chocolate agar (anaerobic). Any organism grown was identified using standard biochemical tests, followed by antimicrobial susceptibility testing (Clinical and Laboratory Standard Institute guidelines).^18^ The timing of the reporting of culture results was noted (standard: within 24–48 h), as was timing of the reporting of the resistance pattern (standard: <72 h).

### Study population/participants

All neonates admitted to the NICU of NMCTH, Biratnagar, Nepal, between 1 January and 31 December 2019 were ascertained from hospital records. We identified cases of suspected sepsis using criteria adapted from All India Institute of Medical Sciences Protocols in Neonatology (Table 1).^19^

**TABLE 1:** Definition of neonatal suspected sepsis

Clinically suspected neonatal sepsis: neonates with clinically suspected sepsis having a constellation of signs and symptoms that can include fever, poor feeding, respiratory distress, cyanosis, cold clammy skin, tachycardia, seizures, hyperreflexia, jaundice and temperature or blood pressure instability; all neonates in whom sepsis was suspected were included in the study
Confirmed neonatal sepsis: defined as the presence of a clinically significant pathogen isolated from blood cultures with associated signs and symptoms

### Sources of data and data collection

From the clinical and laboratory records and discharge summary, we collected sociodemographic characteristics, risk factors associated with sepsis, microorganism growth and antibiotics susceptibility patterns, and hospital exit outcome in relation to septicaemia treatment using a structured pro forma.

### Data validation

All data were double-entered and validated using Epi-Data database v3.1 for entry (EpiData Association, Odense, Denmark). Data were analysed using SPSS v21 (IBM SPSS, Armonk, NY, USA).

### Analysis and statistics

The number and proportion of neonates admitted for suspected septicaemia and with samples sent for blood culture were calculated, along with median and inter-quartile ranges for the time taken for laboratory procedure, timing of changes in regimen from the issuing of the laboratory report and duration of empirical treatment. Poisson regression modelled any association between the demographic and clinical characteristics, hospital exit outcome, potential confounders and antibiotic resistance. The level of significance was set at *P* < 0.05.

### Ethics approval

Permission for the study was obtained from Institutional Review Committee (IRC), Nobel Medical College and Teaching Hospital (NMCTH) (Ref: IRC-NMCTH 162/076 of 29/01/2020). The study was also approved by the Ethics Advisory Group of the International Union Against Tuberculosis and Lung Disease, Paris, France (EAG number: 08/20, date 2020-02-05). As this was a record review with no patient identifiers, the issue of informed patient consent did not apply.

## RESULTS

### Social and clinical characteristics

In total, 200 relevant cases were identified. Around two thirds of these cases were inborn (*n* = 146, 73.0%), male (*n* = 130, 65.0%), born at full-term gestation (*n* = 140, 70.0%) and with normal birth weight (*n* = 141, 70.5%).

### Sepsis

Sepsis was clinically suspected in 177 (88.5%) cases. More than two thirds of them presented with features of early onset sepsis, while many also had a range of comorbidities. Most of the sepsis cases were inborn, admitted within first week of life, males, born at term or of normal birth weight (Table 2). Among inborn neonates, early onset sepsis (*n* = 100, 88.7%) was more common, whereas late onset sepsis (*n* = 38, 71.7%) was more common in outborn neonates.

**TABLE 2:** Demographic and clinical characteristics of neonates with suspected sepsis admitted to the NICU at Nobel Medical College and Teaching Hospital, Biratnagar, Nepal, January–December 2019

Characteristics	*n*	% (column percentages)
Total	177	88.5
Sociodemographic characteristics		
Age, weeks		
1	146	82.4
2	11	6.2
3	9	5.0
⩾4	11	6.2
Sex		
Male	113	63.8
Female	64	36.1
Delivery site^*^		
In-born	124	70.0
Out-born	53	29.9
Birth weight, kg		
Normal (>2.5)	126	71.1
Low birth weight (2.5–1.5)	39	22.0
Very low birth weight (1–1.5)	12	6.7
Gestational age, weeks		
Term (37–42)	123	69.4
Preterm ( <37)	51	28.8
Post-term ( >42)	3	1.6
Class of sepsis (physician-defined)		
Early onset sepsis	124	70.0
Late onset sepsis	51	28.8
Not recorded	2	1.1
Comorbidities		
Present	30	16.9
Absent	69	38.9
Not recorded	78	44.0
Culture report		
Sterile	117	66.1
Positive	52	29.3
Not available	8	4.5
Change in regimen		
Yes	36	20.3
No	141	79.6
Change in regimen among positive culture result		
Yes	36	69.2
No	16	30.7
Hospital exit outcome		
Improved	170	96.0
Leave against medical advice	7	3.9

^*^ Patients admitted from another hospital, primary health care centre, health posts and home deliveries from same or different province.

NICU = neonatal intensive care unit.

Out of the total samples tested (*n* = 177), 52 (29.3%) were culture-positive, of which in 36 (69.2%) the empirical antibiotic regimen was revised as per blood culture sensitivity pattern. Almost all cases admitted with sepsis improved with the given regimen (Table 2). Gram-negative bacterial infection was the main cause of septicaemia among neonates aged 1 week at admission, predominantly affecting males, inborn babies and those with low birth weight. Gram-negative sepsis was more common in babies of all gestational ages, and was most common in both early and late onset neonatal sepsis (Table 3).

**TABLE 3:** Demographic and clinical characteristic according to culture positivity in neonates with suspected sepsis admitted to the NICU at Nobel Medical College and Teaching Hospital, Biratnagar, Nepal, January–December 2019

	Total *N*	Gram-positive bacteria		Gram-negative bacteria	
	
		*n*	% (Row percentage)	*n*	% (Row percentage)
Total	52	7	13.4	45	86.5
Age, weeks					
1	45	6	13.3	39	86.6
2	3	0	0.0	3	100
3	2	1	50.0	1	50.0
⩾4	2	0	0.0	2	100
Sex					
Male	38	2	5.2	36	94.7
Female	14	5	35.7	9	64.2
Delivery site					
Inborn	40	6	15.0	34	85.0
Outborn	12	1	8.3	11	91.6
Birth weight					
Low birth weight (2.5–1.5)	6	2	33.3	4	66.6
Very low birth weight (1–1.5)	4	2	50.0	2	50.0
Normal	42	3	7.1	39	92.8
Gestation age, weeks					
Preterm ( <37)	12	3	25.0	9	75.0
Term (37–42)	38	4	10.5	34	89.4
Post-term ( >42)	2	0	0.0	2	100
Class of sepsis					
Early onset sepsis	39	5	12.8	34	87.1
Late onset sepsis	13	2	15.3	11	84.6
Not recorded					
Comorbidities					
Present	4	1	25.0	3	75.0
Absent	45	6	13.3	39	86.6
Not recorded	3	0	0.0	3	100

^*^ Patients admitted from another hospital, primary health care centre, health posts and home deliveries from same or different province. NICU = neonatal intensive care unit.

### Antimicrobial resistance

*Pseudomonas* predominated, followed by coagulase-negative *Staphylococcus* (Table 4). Overall, 9 (17.0%) culture-positive cases of septicaemia were resistant to the AWaRe (Access, Warch, Reserve) group of antibiotics. The most common isolate was sensitive to Access and Watch group antibiotics, whereas occasional resistance was observed with the Reserve group of drugs (Table 5).

**TABLE 4:** Culture-positive pattern among neonates with suspected sepsis admitted to the NICU at Nobel Medical College and Teaching Hospital, Biratnagar, Nepal, January–December 2019

Organisms (*n* = 71)	Culture-positive	

	*n*	%
Pseudomonas	40	78.4
Coagulase negative staphylococcus	12	23.0
Klebsiella	5	9.8
Citrobacter	5	9.8
*Escherichia coli*	4	7.8
Acinetobacter species	2	3.9
Enterococcus	3	5.8
Others^*^	0	0.0

^*^ Includes *Neisseria gonorrhoea*.

NICU = neonatal intensive care unit.

**TABLE 5:** Antimicrobial resistance of the isolated microorganism among neonates with suspected sepsis admitted to the NICU at Nobel Medical College and Teaching Hospital, Biratnagar, Nepal, January–December 2019

Organism	Isolates *N*	Drug *n* (%)	AWaRe Group of antibiotics^34^
Pseudomonas	40	Imipenem: 1 (2.5)	Reserve
		Linezolid: 1 (2.5)	
Coagulase-negative Staphlococcus	12	—	
*Citrobactor* spp.	5	Ampicillin: 3 (60)	Access
		Amikacin: 2 (40)	Watch
		Gentamicin: 2 (40)	
		Cefotaxime: 3 (60)	
		Azithromycin: 1 (20)	
*E. coli*	4	Amikacin: 3 (75)	Access
		Ampicillin: 2 (50)	
		Cefotaxime: 2 (50)	
		Gentamicin: 1 (25)	
*K. pneumoniae*	3	Ampicillin: 1 (20)	Access
		Cefotaxime: 1 (20)	
		Gentamycin: 1 (20)	
Enterococcus	3	—	
Acinectobactor	2	Ampicillin: 1 (50)	Access

NICU = neonatal intensive care unit.

### Turnaround times

The median turnaround time for laboratory culture was 3 days (range 2.0–4.0). The median time to change empirical therapy after a positive culture and sensitivity report was 3 days (range 3.0–6.0).

### Associations with culture-proven sepsis

Of culture-positive cases, two characteristics showed a statistically significant effect on positive culture results: presence of comorbidities reduced the risk of a positive culture (adjusted relative risk [aRR] 0.23, 95% confidence interval [CI] 0.09–0.57), while being post-term increased the risk (aRR 1.33, 95% CI 1.05–1.69) (Table 6). None of the factors which affected the risk of a positive culture were significantly associated with single drug resistance.

**TABLE 6:** Factors associated with culture positivity among neonates with suspected sepsis admitted to the NICU at Nobel Medical College and Teaching Hospital, Biratnagar, Nepal, January–December 2019

Characteristics	Total *n*	Culture-positive		RR	*P* value^*^	aRR	95% CI

		*n*	%				
Total		52	29.3				
Age, weeks							
1	146	45	30.8	1.69	0.41	1.32	0.53–3.31
2	11	3	27.2	1.50	0.61	1.41	0.46–4.27
3	9	2	22.2	1.22	0.82	0.93	0.24–3.61
⩾4	11	2	18.1	Reference		Reference	
Sex							
Male	113	38	33.6	1.54	0.11	1.27	0.80–2.00
Female	64	14	21.8	Reference		Reference	
Delivery site^†^							
Inborn	124	40	32.2	Reference		Reference	
Out-born	53	12	22.6	0.70	0.21	0.88	0.48–1.62
Birth weight, kg							
Normal (>2.5)	126	42	33.3	Reference		Reference	
Low birth weight (2.5–1.5)	39	6	15.3	0.46	0.05	0.77	0.36–1.62
Very low birth weight (1–1.5)	12	4	33.3	1	1.00	1.17	0.39–3.49
Gestational age, weeks							
Term (37–42)	123	38	30.8	Reference		Reference	
Preterm (<37)	51	12	23.5	0.76	0.34	0.78	0.43–1.42
Post-term (>42)	3	2	66.6	2.16	0.07	1.33	1.05–1.69
Class of sepsis (physician defined)							
Early onset sepsis	124	39	31.4	1.23	0.44	0.76	0.43–1.36
Late onset sepsis	51	13	25.4	Reference		Reference	
Comorbidities							
Yes	30	4	13.3	0.20	0.00	0.23	0.09–0.57
No	69	45	65.2	Reference		Reference	

^*^P < 0.05, statistically significant.

^†^ Patients admitted from another hospital, primary health care centre, health posts and home deliveries from same or different province. NICU = neonatal intensive care unit; RR = risk ratio, aRR = adjusted RR; CI = confidence interval.

### Hospital exit outcomes

Of culture-positive cases, 94.2% (*n* = 49) improved, but 3 (5.8%) left against medical advice.

## DISCUSSION

### Demographic and clinical characteristics

In a tertiary NICU in Nepal, over 88% of cases were diagnosed with clinically suspected sepsis, of which two thirds presented with features of early onset sepsis. However, under one third had culture-proven septicaemia, mainly caused by Gram-negative bacteria. Risk factors for sepsis were not identified; AMR was noted but not statistically associated with risk factors or exit outcome of the neonate from NICU. The presence of comorbidities was associated with a reduced risk of being culture-positive.

### Neonatal sepsis

Clinical sepsis was confirmed in under one third of neonates. Our confirmation rate is in the middle of other studies in Nepal and elsewhere.^20^–^25^ Empirical treatment may have started before blood was taken for culture or before admission to the NICU. Early use of antibiotics before taking blood for culture is known to lead to sterile culture reports. This needs further investigation, possible remedial action within the NICU and obstetric unit and consideration if the integrated preventive and protective measures against infections have failed, and why, or if there are other factors influencing the high rate of unconfirmed sepsis.

Among suspected sepsis cases, more than two thirds presented with features of early onset sepsis, which is higher than other nearby reports.^24^–^26^ Probable explanations include 1) most of our new-borns were inborn with subsequent early identification of clinical sepsis and prompt admission to NICU; and 2) the causative agents for early onset sepsis were acquired from the mother during birth.^1^ Further investigation into the possible acquisition of sepsis at birth is needed to protect vulnerable new-borns.

The predominance of male new-borns has been reported elsewhere.^2^,^24^,^27^,^28^ This may be due to the prevalent societal preference given to males in seeking medical care.^26^ However, males may also be vulnerable to neonatal sepsis due to an X-linked immune regulatory gene factor.^29^ With improved confirmation of sepsis, it would be possible to investigate this sex difference and begin to counter any bias against female infants.

We found that comorbidities (meconium aspiration syndrome, bowel obstruction, etc.) significantly reduce the risk of having a positive culture. However, there was a lot of missing data, possibly biasing this unexpected finding. Comorbidities could be expected to predispose to the overuse of broad-spectrum antibiotics, leading to sterile cultures. In addition, NMCTH receives referral cases, many with comorbidities and previous treatment, from different parts of Nepal.

There are at least two approaches to sterile cultures. First, any use of empirical antibiotics needs to be kept to a minimum by more rapid turnaround times from the laboratory, or (preferably) quick and accurate bedside point-of-use diagnostic tests for sepsis, thereby reducing the need for empirical treatment, as in other infections.^30^,^31^ Second, enhanced professional and continuing education of all health care providers, wherever situated, would ensure proper understanding and awareness regarding antimicrobials, including the collection of blood samples for culture before starting empirical therapy.

### Antimicrobial resistance profile

Gram-negative organisms are the most common agent causing neonatal sepsis,^2^,^25^,^32^ as we were able to confirm (Table 4). However, few studies have reported Gram-positive bacterial septicaemia among neonates in the hospital setting. Among these, methicillin-resistant *S. aureus* was a critically important pathogen in the NICU population, associated with both endemic and epidemic infections.^20^,^28^

Host and bacterial properties unite to cause sepsis in newborns. The organism varies according to the environmental conditions of the hospital, e.g., contamination during sampling, care of both in-born and out-born cases, and surgical cases mixed with medical cases.^26^

Contamination during sampling or during birth from the mother’s birth canal could account for coagulase-negative *Staphylococcus*. The latter is more life-threatening, and further investigation to distinguish between the two sources is important. In our study, the predominance of *Pseudomonas* suggests transmission of infection from healthcare workers to patients via contaminated hands. Accordingly, re-enforcement of infection control guidelines and education of healthcare staff are important. Our study further prompts the need for the implementation of the WHO AMR action plan in Nepal.

The most common antibiotic resistance was in *Pseudomonas*, unlike a previous report from Nepal,^26^ which showed that Gram-negative organisms were resistant to ampicillin. Other isolates in our study were, not surprisingly, resistant to first-line and second-line therapy. A potential way to curb this would be increased and timely surveillance of antibiotic sensitivity patterns and revision of policies regarding empirical therapy based on AMR, along with the implementation of the aforementioned point-of-use diagnostic testing for sepsis.

### Turnaround time for samples and empirical therapy

The longer the administration of broad-spectrum antibiotics, the greater the progression of severe sepsis to septic shock, with increased mortality.^33^ The median turnaround time taken for laboratory culture plus the median time to changing to an appropriate antibiotic regime means that it is possible for a septic neonate to wait ⩾6 days for a necessary change to appropriate treatment. This again indicates the need for rapid diagnostic tests.

### Strengths

The study is one of the few which report AMR and the turnaround time for the reporting of culture results and consequent change in antibiotic management.

### Limitations

The small numbers of positive blood cultures meant that none of the risks likely to be associated with resistance to at least one antibiotic or to a hospital exit outcome were fully assessed. Second, as critically ill patients who left against medical advice were likely to have died, our estimate of the mortality rate may have been an underestimate.

## CONCLUSION

*Pseudomonas* was the most common isolate in culture-proven sepsis. Gram-negative bacilli were susceptible to Access and Watch group antibiotics, whereas, occasional resistance was observed with last resort (Reserve) antibiotics. Rapid diagnostic tools for sepsis, which would also identify AMR and conform to accepted standards and guidelines, are urgently needed in NICUs to enable the antibiotic regime to be appropriate from the earliest time possible.

As sepsis is a major health risk for neonates, a standardised national and global surveillance programme collecting neonatal AMR data, including risk factors and clinical outcomes, is critical. This would enable benchmarking, the identification of remedial risks, the design and implementation of successful interventions, and allow review of NICU programmes.
